# The META tool optimizes metagenomic analyses across sequencing platforms and classifiers

**DOI:** 10.3389/fbinf.2022.969247

**Published:** 2023-01-06

**Authors:** Robert A. Player, Angeline M. Aguinaldo, Brian B. Merritt, Lisa N. Maszkiewicz, Oluwaferanmi E. Adeyemo, Ellen R. Forsyth, Kathleen J. Verratti, Brant W. Chee, Sarah L. Grady, Christopher E. Bradburne

**Affiliations:** ^1^ Applied Physics Laboratory, Johns Hopkins University, Laurel, MD, United States; ^2^ Division of General Internal Medicine, Johns Hopkins School of Medicine, Baltimore, MD, United States; ^3^ Armstrong Institute for Patient Safety and Quality, Johns Hopkins School of Medicine, Baltimore, MD, United States; ^4^ McKusick-Nathans Department of Genetic Medicine, Johns Hopkins School of Medicine, Baltimore, MD, United States

**Keywords:** metagenomics, metagenomic classification, testing and evaluation, NGS, oxford nanopore, illumina, classifier

## Abstract

A major challenge in the field of metagenomics is the selection of the correct combination of sequencing platform and downstream metagenomic analysis algorithm, or “classifier”. Here, we present the Metagenomic Evaluation Tool Analyzer (META), which produces simulated data and facilitates platform and algorithm selection for any given metagenomic use case. META-generated *in silico* read data are modular, scalable, and reflect user-defined community profiles, while the downstream analysis is done using a variety of metagenomic classifiers. Reported results include information on resource utilization, time-to-answer, and performance. Real-world data can also be analyzed using selected classifiers and results benchmarked against simulations. To test the utility of the META software, simulated data was compared to real-world viral and bacterial metagenomic samples run on four different sequencers and analyzed using 12 metagenomic classifiers. Lastly, we introduce “META Score”: a unified, quantitative value which rates an analytic classifier’s ability to both identify and count taxa in a representative sample.

## Introduction

Since its inception, the field of metagenomics has proven to be one of the most challenging arenas of genomics research. Microbial communities are often dynamic, and the countless tools available for characterization all present their own strengths and weaknesses. Experimental design choices are frequently made with reagent cost, availability, and protocol ease in mind, with less emphasis placed on a thorough understanding of the limitations of a particular sequencer, metagenomic classification algorithm, reference database, or the complexity of the sample itself. Here, we introduce the Metagenomic Evaluation Tool Analyzer, or “META”, as a solution for predicting and testing the best approach for a given metagenomic experiment (Table 1). Users are able to use this modular and easily updateable software to select, simulate, and compare the performance of different sequencer/classifier combinations towards more efficient use of available wetlab resources.

**TABLE 1:** META features and rationale.

META feature	Implemented by	Enables	Achieves
Modular	Dockers	Futureproofing *via* ease of updating	Recent version-controlled algorithms
		Ease of expanding as new classifiers are published	
		Classification tools with diverse dependancies	
Down-scalable	Choice of tools and database size	Broader range of deployment options based on available hardware	Pre-tested, deployable bioinformatics
		Selection of optimal pipeline at large scale, and deployment on laptop	
Simulation	InSilicoSeq and DeepSimulator	Read generation from a single source across sequencers	Downselection of sequencer and analysis
		Evaluation and selection of analysis algorithms without costly sequencing	
Visualization	D3.js	Interactive visualizations	Ease of use and interpretation
		Comparison of classifiers based on simulated or real reads	
Resource estimation	Tracking of compute time, RAM usage	Data driven hardware assessment	Optimal hardware utilization
		Estimate of analysis time	
Optimization	User	Selection of the best classifier based on user needs	Validated, deployable bioinformatics
		GUI-enabled bioinformatic analysis	
		Selection of optimal pipeline at large scale, and deployment on laptop	

### Need and existing work

The difficulty inherent to the rapid and accurate classification of metagenomic sequencing data is a problem many researchers have attempted to solve, each publishing their own classification tools and comparing their performance against peer algorithms and/or third-party datasets (Ye et al., 2019). New algorithms are introduced on a constant basis, while older ones are updated periodically. The steady progression of sequencing platforms, can also make it difficult for an end user to make an educated decision regarding the optimal sequencer/classifier pairing. META was developed to help users select from an ever-evolving landscape of emerging metagenomic classifier tools (Park and Kim, 2016). As far as the authors can tell, only two other platforms have the potential to exist in the same application space as META: Galaxy and the Open-Community Profiling Assessment tooL (OPAL). However, while galaxy could provide the foundation for side-by-side comparisons, there are no integrated evaluation tools (Afgan et al., 2018), and while OPAL provides more composition-level metrics for performance comparison, it lacks interactive data visualizations or table filtering options for classifier output (Meyer et al., 2019). Additionally, resource metrics such as run times and peak memory usage aren’t readily reported in the native installation state of OPAL, which requires a separate step of converting it to a Biobox Docker image. META provides all of these utilities by default, comparing the performance of more than ten classifiers and utilizing read simulators for the Illumina (MiSeq and iSeq instruments) and Oxford Nanopore Technologies (R9 and FLG flowcells) sequencing platforms (Li et al., 2018; Gourlé et al., 2019). META is openly available and can be accessed at the following GitHub URLs: https://github.com/JHUAPL/meta-system, and https://github.com/JHUAPL/meta-simulator. For users already running Docker, containers may be accessed at https://quay.io/repository/jhuapl/meta_system, and https://quay.io/repository/jhuapl/meta_simulator.

### Simulated versus real world sequencing modes

**FIGURE 1:**
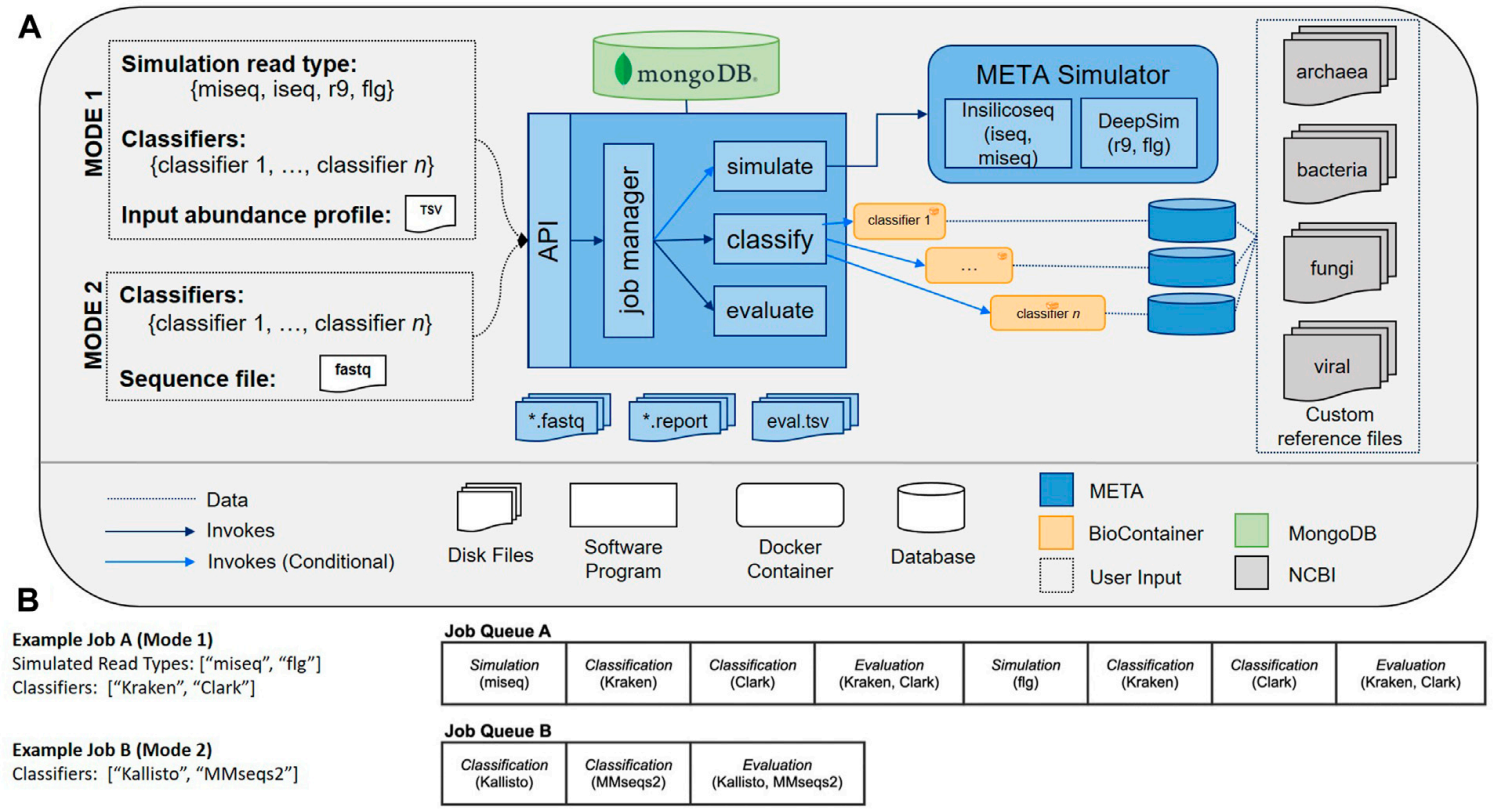
**(A)** META system architecture. The META system supports two evaluation modes: Mode 1 (*in silico* generated reads), and Mode 2 (real-world generated reads). Mode 1 enables classifier output to be compared to “ground truth” abundance profiles as supplied by the user. Reads can be generated using multiple sequencing platform simulators. After selecting which classifiers to evaluate and submitting a job, the system metadata is tracked in a MongoDB database while each selected classifier is run in series. **(B)** Sample META analytics workflows. Workflows are generated upon user request, and include serially-run simulation, classification, and evaluation modules.

The META bioinformatics analysis pipeline enables the direct and simultaneous comparison of metagenomic abundance profiles derived from multiple classifiers using a set of *in silico* (simulated), or “real-world” sequencing reads (Figure 1). In this way, the system supports two modes of classifier comparisons. “Mode 1” enables the multi-classifier analysis of an *in silico* generated read set using sequencing read simulators (specifically InSilicoSeq (Gourlé et al., 2019) for Illumina platforms, and DeepSim (Li et al., 2018) for Nanopore) that produce data reflecting a user-defined abundance profile. Results are reported using three metrics: Area under the precision recall curve (AUPRC) (Ye et al., 2019), the Euclidean distance between the user-defined abundance profile and the predicted abundance profile (L2) (Ye et al., 2019), and the *metascore*, which is a normalized ratio of these two values. The AUPRC is a measure of how well individual taxa are identified and is more sensitive to low-abundance taxa, while the L2 metric is a measure of how reliably the abundance is calculated, meaning it is more sensitive to high abundance taxa. The meta score combines these values and provides a simple measure of how well taxa are both identified and counted in a given sample. A complete description of the L2, AUPRC, and meta score can be found in the materials and methods.

“Mode 2” enables a comparison of output from multiple classifiers when the input consists of any FASTQ file, including those generated from real wet-lab experiments. Used in tandem, these two modes can help select the best sequencing and analysis approach prior to, and following, the experimental design phase.

### Classifiers, modularity, and standardization

The initial release of META contains a total of twelve classifiers (Table 2). There are seven k-mer-based algorithms: Bracken (Lu et al., 2017), CLARK (Ounit et al., 2015), Kraken (Wood and Salzberg, 2014), Kraken2 (Wood et al., 2019), KrakenUniq (Breitwieser et al., 2018), Mash (Ondov et al., 2019), and MMseqs2 (Steinegger and Söding, 2017). The remaining five classifiers use alignment-based algorithms: Centrifuge (Kim et al., 2016), HS-BLASTN (Ying et al., 2015), Kallisto (Bray et al., 2016) DIAMOND (Buchfink et al., 2015), and Kaiju (Menzel et al., 2016). The former three utilize nucleotide sequence databases, while the latter two utilize protein sequence databases.

**TABLE 2:** Metagenomic classification tools and associated metrics that are available in initial META public release. Custom database build size refers to the custom databases generated from the normalized set of references (units are gigabytes (GB) unless otherwise noted). Average peak memory usage and run times were derived from a total of 16 Mode 1 jobs of varying metagenomic composition (FASTQ file size averaging approximately 1.0 GB), and using the custom built databases associated with each classifier. Descriptions of the versions of each classifier run in this test can be found at https://github.com/JHUAPL/meta-system

No.	Classifier	Synopsis	Custom database build size (GB)	Average peak memory usage (GB)	Average runtime (s)
1	Bracken	k-mer based abundance estimation from raw reads	72 KB	0.210	0.04
2	Centrifuge	alignment based on BWT and FM-indexing schemes	30	2.717	14.38
3	CLARK	k-mer based species or genus level classification	255	9.901	0.04
4	DIAMOND	alignment based, SW against protein database	24	0.935	145.47
5	HS-BLASTN	An accelerated MegaBLAST search tool	274	28.282	74.08
6	Kaiju	alignment based on BWT and FM-indexing of protein sequences	26	2.672	14.63
7	Kallisto	alignment based, pseudoalignment procedure	15	2.524	0.05
8	Kraken	k-mer based, exact alignment	45	0.240	4.95
9	Kraken2	k-mer based, exact alignment, and translated search mode	32	0.205	0.32
10	KrakenUniq	k-mer based, exact alignment with smaller memory requirements	819	0.245	71.05
11	Mash	k-mer based, locality sensitive hashing	16 KB	0.158	0.64
12	MMseqs2	k-mer based filtering with ungapped then ungapped alignments	651	0.373	0.07

META is modular, making extensive use of Docker architectures *via* Bio containers, to facilitate rapid updating or addition of new classification tools as they become available (Merkel, 2014; BioContainers, 2020). The system also can accommodate currently-available common standards from the Global Alliance for Genomics & Health (GA4GH) to improve interoperability, security, privacy, data visualization, and compatibility (Merkel, 2014; GA4GH, 2020). Using these community-developed standards allows the system to have immediate utility across a wide range of scenarios and use cases.

### Test cases and simulated versus real world evaluation

**FIGURE 2:**
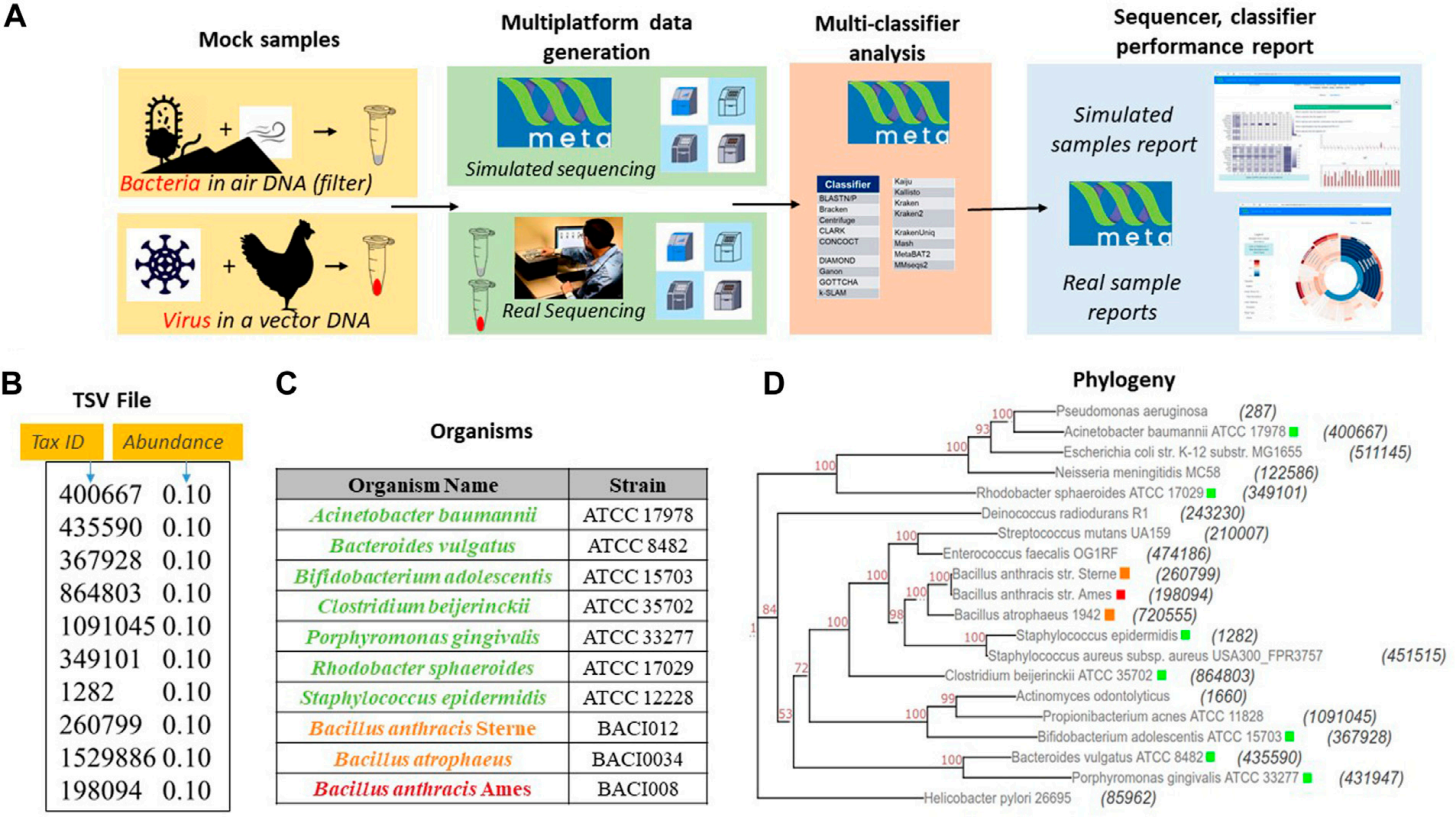
Sample use-cases test META capabilities. **(A)**. Sequencing results from two mock metagenomic DNA communities are produced in the lab and mimicked *in silico*. Results using different classifiers are compared to the known sample content to identify the best performing sequencer/classifier combination. **(B)**. Mode 1 user-generated TSV file for use case 1 mimicking a community found on an air filter. **(C)** Detailed components of community includes a threat agent (*B. anthracis*), two near neighbor organisms (*B. anthracis* Sterne and *B. atropheus*), and seven background environmental organisms selected from an American Type Culture Collection (ATCC) 20 strain Even mix genomic sample (Cat. # MSA-1002). **(D)** Phylogenetic distance between each component of use case 1 and the community in the 20 strain even mix sample. The tax ID is shown in parenthesis and the color code corresponds to the organism list in Figure 2.

To test the utility of META, our group designed a set of two experiments using real world samples sequenced on multiple platforms (Figure 2A). Use case 1 was designed to represent an environmental surveillance scenario, in which an aerosol sample, containing the human pathogen *Bacillus anthracis* and two non-pathogenic near-neighbors, was collected in a background of seven common environmental bacteria (Figures 2A–D). Use case 2 was designed to represent a sample collected from an animal infected with a potential human pathogen. *Vaccinia* virus, a simulant for smallpox, was used as the pathogen, and the chicken cells used to propagate the virus represented the infected animal (Figure 2A). All samples were sequenced on both Illumina and Oxford Nanopore platforms and *in silico* reads were generated on the same platforms. FASTA and FASTQ files for both wetlab and *in silico* simulated samples were then run through META’s classifier algorithms, and reports were generated describing performance.

## Results

### Survey and down-selection of classifiers for initial release

A literature search identified eighty-one metagenomic classifiers that were subsequently down-selected for inclusion in the initial release of the META system based on seven critical attributes (described in detail in Methods). Eighteen classifiers remained after down-selection criteria, and twelve were successfully implemented (Table 2). Classifiers used a range of algorithm strategies and custom reference databases.

### Use-case 1

Mode 2 was utilized to evaluate the real-world data derived from experimental samples developed during use case generation. Classifiers were ranked by metascore for each read type (Illumina and ONT-generated reads) using the known spiking concentration for each component of the sample. Corresponding *in silico*-generated reads were then processed using Mode 1. Accurate strain and species identifications were prioritized in all analyses as this facilitated the differentiation of pathogens from background organisms.

**FIGURE 3:**
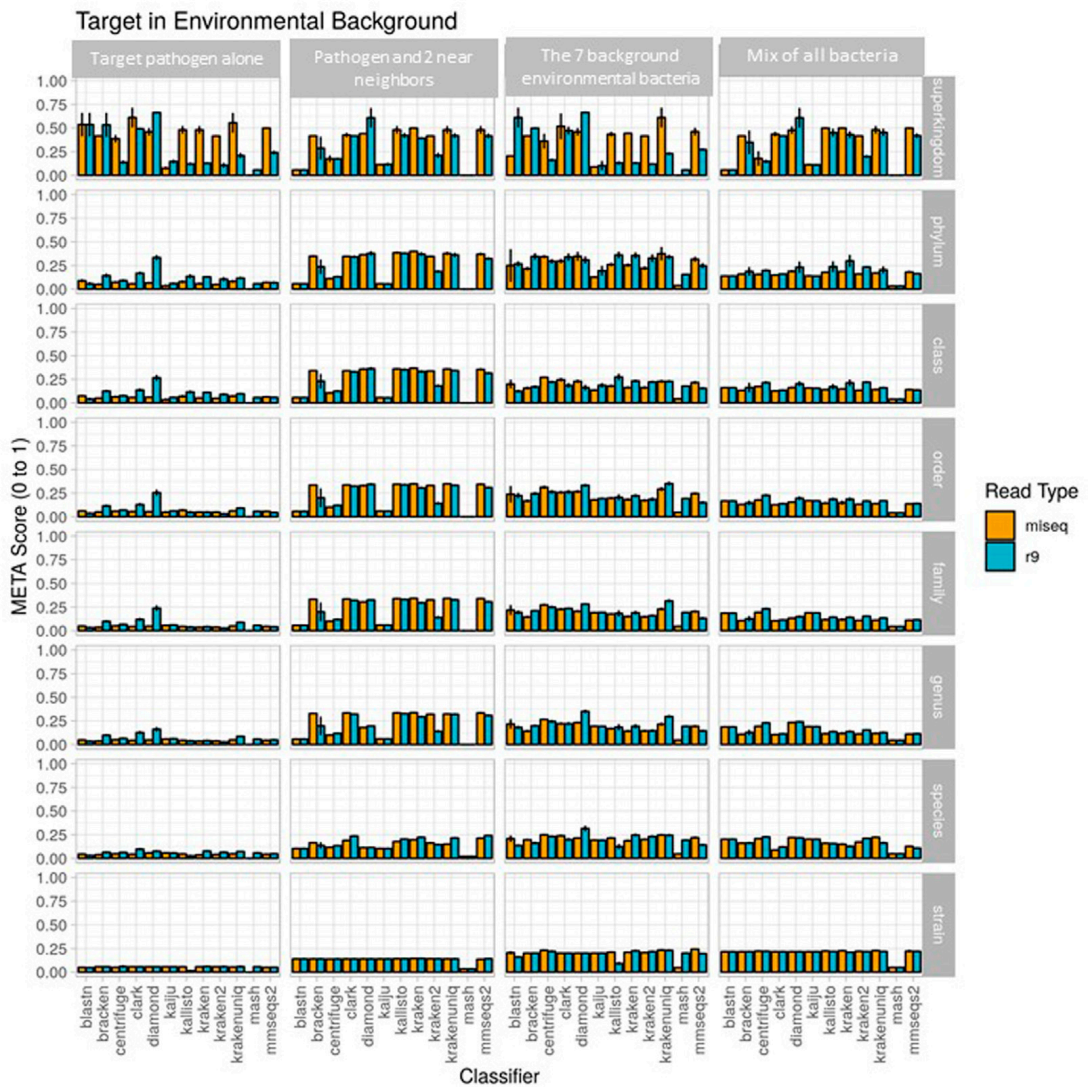
Use case 1 output. Classifier performance from *in vitro* data sets is illustrated using the metascore metric from super kingdom to strain taxonomic ranking. All twelve available classifiers were run using data generated from Illumina MiSeq and the ONT R9 flowcell. *N*=3. Further visualization of these results can be found at https://meta-results.jhuapl.edu.

The metascores for each sequencer/classifier pairing for use case 1 are shown in Figure 3. (L2 and AUPRC scores are displayed in Supplementary Figures S1, S2). Separate analyses were run on 4 sample types: the pathogen alone, the pathogen and two near-neighbors, the seven background environmental bacteria, and all ten components together. Most classifiers scored similarly within each sample type and for each read type at the strain rank, with Mash having the lowest metascore in all cases. In general, the average metascore among all sequencer/classifiers pairings decreased as the total number of known organisms in a sample decreases. This isn’t unexpected, as the ability to discriminate between the total reads available become more difficult when the available reads in a sample are nearly identical. For the “mix of all 10” organism samples, Kraken Unique achieved the highest metascore for the Illumina (MiSeq) read type, and Centrifuge the highest score for the ONT (R9 flowcell) read type.

Classifier performance for samples containing multiple organisms could also be determined by identifying 1) how many of the expected organisms were detected at any level, and 2) how closely their respective abundance profiles matched the known spiking concentrations. While no classifier would be expected to identify all ten organisms, as the *Bacillus atrophaeus* subsp. *globigii* taxID was purposefully omitted in the custom reference database, Kraken, Kraken2, and HS-BLAST were the best performers, identifying all of the remaining 9 taxIDs when analyzing both Illumina and Nanopore reads (Table 3, columns two and three).

**TABLE 3:** Deviation of pathogenic target organism abundance from theoretical known value in use case 1. Values are representative of samples containing all 10 possible components at equal genome copy input. *N*d= not detected.

	Number of expected taxids identified in output (max = 9)		Calculated *B. anthracis* abundance (theoretical max = 10%)	
	Illumina	ONT	Illumina	ONT
Bracken	1	1	nd	nd
Centrifuge	5	4	nd	nd
CLARK	1	1	nd	nd
DIAMOND	1	1	nd	nd
HS-BLASTN	9	9	19.20	10.65
Kaiju	1	0	nd	nd
Kallisto	9	7	0.09	nd
Kraken	9	9	0.22	0.34
Kraken2	9	9	0.10	0.11
KrakenUniq	9	8	nd	nd
Mash	0	0	nd	nd
MMseqs2	9	8	nd	nd

The ability of the classifiers to identify the pathogenic component of the 10-organism samples was of particular interest. The three classifiers listed above successfully identified BA in Nanopore data sets, and the same three, plus Kallisto, identified BA from Illumina data sets. Theoretically, the *B. anthracis* read abundance should fall at approximately 10% for all of these sequencer/classifier pairs, but of the four classifiers that identified the pathogen at all using Illumina results, three were below 1%, while HS-BLASTN significantly over-represented its presence at >19%. The three classifiers identified the *B. anthracis* taxID from ONT results, with two of the three classifiers that identified *B. anthracis* using Nanopore data sets significantly under-represented abundance (<1%) with only HS-BLASTN coming within 7% the expected value.

With respect to computer resource utilization, run times and peak memory usage varied widely based on the selected sequencer and classifier combination. Generally, though, no clear trend related these metrics with the metascore values, suggesting that users couldn’t base their selection on these predictions alone. Full interactive reports of the individual sequencing runs that generated this data are available for exploration here (https://meta-results.jhuapl.edu).

### Use case 2

**FIGURE 4:**
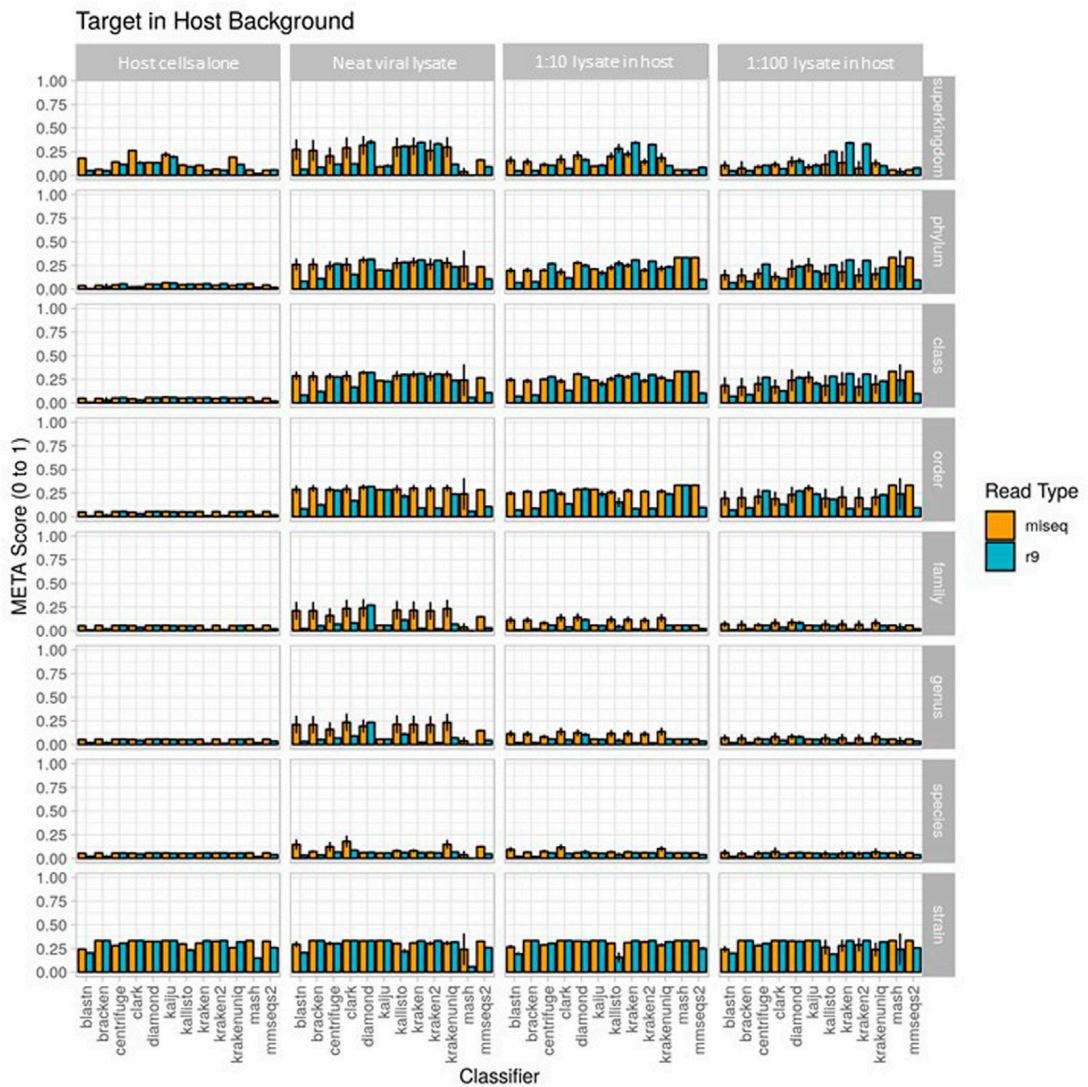
Use case 2 output for 1) host cells alone, 2) extraction 1, a neat viral lysate), 3) spike 1, a 1:10 dilution of viral lysate in host background), and 4) spike 2, a 1:100 dilution of viral lysate in host background. Classifier performance from *in vitro* data sets is illustrated using the metascore metric from superkingdom to strain taxonomic ranking. All twelve available classifiers were run using data generated from Illumina MiSeq and the ONT R9 flowcell. *N*=3. Further visualization of these results can be found at https://meta-results.jhuapl.edu.

Results from the second use case are displayed in Figure 4 (metascores) and Supplementary Figures S3, S4 (AUPRC) and (L2). For the Illumina data, the classifier achieving the highest metascore for “host”, “extraction1”, “spike1” and “spike2” was Mash (0.0572), CLARK (0.1787), CLARK (0.1165), and CLARK (0.0710), respectively. For the ONT data, the classifier achieving the highest metascore was DIAMOND (0.0571), CLARK (0.0861), DIAMOND (0.0673), and DIAMOND (0.0611), respectively (Figure 4). With respect to computer resource utilization, and only using those of the “spike2” sample, using the simulated Illumina read set CLARK was ranked 2nd for runtime at only .42 s, however it was ranked 11th for peak memory usage at over 118 GB RAM. Using the simulated ONT read set from the same sample profile, DIAMOND was ranked 11th for runtime at 345 s, and 7th for peak memory usage at only 8.6 GB RAM. Full interactive reports are available for exploration here (https://meta-results.jhuapl.edu).

A note here on the effects of this use-case (a single target of interest) on the value of AUPRC and L2. The AUPRC is very low across all taxonomic ranks for each sample type, implying there are many false positive classifications which is likely due to the fact that there is only a single “ground truth” tax ID for these samples (the spiked organism). The L2 metric is much more variable across both taxonomic rank and sample type, and is actually closer to the ground truth profile at the stain rank compared to the higher ranks. This is somewhat counter-intuitive as one would assume that a more specific classification (e.g., strain) is more difficult than a less specific classification (e.g. species). However, and again since there is only a single “ground truth” tax ID for these samples, even an abundance profile vector containing zeros for all tax IDs is closer to the ground truth than a vector that contains even a single tax ID that isn’t in the ground truth set at any significant abundance. These points coupled with the fact that L2 is in the denominator of the metascore calculation yields the higher strain rank scores seen in Figure 4.

Since the metascore is designed for the evaluation of classifier performance on more traditional metagenomic profiles, it was necessary to also check the deviation from the target ground truth abundance, as was done for the environmental experiment (Table 4). The target genome proportion reflects the target (*Vaccinia* Virus) to host (*Gallus gallus*) ratio in the sample type. The effective ground truth abundance is the abundance used for deviation calculations. This value for sample type 1) is zero, and all classifiers didn’t report it in their output abundance profiles, therefore their deviation is a perfect 0.00%. Sample types 2), 3), and 4) all have a target effective ground truth abundance of one, i.e. the input TSV column 3 for taxID 9,031 (*Gallus gallus*) was set to 0, since it isn’t in the custom reference genome set. As the concentration of the target decreases, the deviation from target ground truth increases. CLARK was identified as the best performer using Illumina read type *via* metascore, and also consistently achieves the lowest deviation from target ground truth abundance for all sample types. Although DIAMOND was identified as the best performer in a majority of sample types using the ONT read type *via* metascore, it had a consistently high deviation from target ground truth abundance. Based on the deviation measure, CLARK, HS-BLASTN, or KrakenUniq may be a better substitute for identifying this target using the ONT read type.

**TABLE 4:** Deviation of pathogenic target organism abundance from theoretical known abundance in use case 2. These show two replicates of these samples, each showing deviation from known abundance for 1) host cells alone, 2) extraction 1, a neat viral lysate, 3) spike of a 1:10 dilution of viral lysate in host background, and i4) spike of a 1:100 dilution of viral lysate in host background. Known abundance was set to 100%, as host genome was not included in the custom reference database. *N*d= not detected.

	Host only	Infected lysate			Host only	Infected lysate		
		Neat	01:10	1:100		Neat	01:10	1:100
Bracken	nd	−82.12%	−83.22%	−92.17%	nd	−84.44%	−94.30%	−99.36%
Centrifuge	nd	−4.80%	−36.93%	−85.23%	nd	−.90%	−85.84%	−98.50%
CLARK	nd	−1.24%	−11.78%	−58.87%	nd	−.70%	−66.78%	−95.15%
DIAMOND	nd	−94.42%	−94.74%	−95.22%	nd	−98.69%	−99.12%	−100.00%
HS-BLASTN	nd	−2.57%	−18.05%	−68.57%	nd	.00%	−77.48%	−97.32%
Kaiju	nd	−100.00%	−100.00%	−100.00%	nd	−100.00%	−100.00%	−100.00%
Kallisto	nd	−77.18%	−79.64%	−90.78%	nd	−82.05%	−90.82%	−100.00%
Kraken	nd	−81.57%	−84.82%	−92.93%	nd	−74.88%	−88.64%	−97.44%
Kraken2	nd	−88.58%	−89.85%	−94.82%	nd	−86.11%	−94.44%	−99.37%
KrakenUniq	nd	−15.22%	−24.55%	−64.05%	nd	−1.40%	−69.38%	−95.80%
Mash	nd	−100.00%	−100.00%	−100.00%	nd	−100.00%	−100.00%	−100.00%
MMseqs2	nd	−2.78%	−53.42%	−92.54%	nd	−37.80%	−88.07%	−99.18%

## Discussion

There is currently no bioinformatics tool that automates 1) the *de novo* simulation of Illumina and Oxford Nanopore Technologies (ONT) metagenomic sequencing data, 2) the simultaneous classification of this *in silico* or real-world sequencing data using multiple algorithms, and 3) provides performance metric information for each algorithm. Here, we present a software package and user interface called META to fill this gap. With META, researchers, can rapidly and inexpensively identify the best classification tool for a specific metagenomics use-case instead of settling on a strategy based on more subjective or qualitative comparisons potentially derived from unrelated data sets. META analysis outputs provide the AUPRC (taxonomy performance) and L2 (abundance performance), and visualizations allow direct performance comparisons between sequencers, classifiers, and metagenomic communities whose complexity can be defined by the user. For the simulation evaluations, we introduce a combined metric, the “metascore”, which provides a simple quantitative measurement of general classifier taxonomy and abundance performance by incorporating both AUPRC to L2 measurements. The metascore is simply a ratio of the AUPRC/L2 that has been normalized from zero to one, with higher metascore ratios being considered better. This score can provide a quick visual way to select the optimal sequencing and bioinformatic analysis approach. For example in the environmental sample use case in which there are no *Bacillus* species (ATCC-only mix of 7), a user interested in the best *Genus* and *Species* overall performance can immediately select Diamond or KrakenUnique, coupled with Oxford Nanopore R9 sequencing for their experiment instead of Illumina, as R9 provides the highest metascore for these classifiers when coupled with R9 sequencing (Figure 3). This mayn’t be readily apparent from observing AUPRC and L2 performance alone, particularly in the case of a single metric under-performing such as AUPRC, or over-performing such as L2 (Supplementary Figures S1, S2). The metascore could eventually be utilized for simple simulation experiments by a user, such as direct comparison to a random classifier, and it offers a quick way to visualize single candidates for bioinformatic algorithm and sequencing approaches. A useful aspect of META is that once sequencing and analysis pipelines are selected, a separate, real-world-mode can be selected and real reads can be run using the same analysis pipeline. The outputs provide data reporting of the real content of a given sample (for examples, see https://meta-results.jhuapl.edu).

META provides feature-rich data visualizations, enabling the user to explore each analysis to the depth that suits their unique needs, eventually coming to their own conclusion about which classifier and read type combination will be most effective. The system is set up to rapidly add new classifiers, or update existing ones, so that a user can determine which sequencer and classifier combination is best suited to their use case, even as the technology continues to change and improve. Applications for this technology are numerous, including environmental monitoring, diagnostics, research, forensics, and industrial processes.

Additional utility would be found if an implementation is made to compare reference databases for classifiers. REFSeq is by far the most utilized reference database, and so most comparisons can be made using it as the initial content base, but META in its current state could still be a useful tool in database content optimization, by running subsets of appropriate databases in a classifier as needed for a particular use case. The next major release of META is expected to include a new mode that will enable reads from a known *in vitro* metagenomic sample dataset to be compared with an expected abundance profile. Assembly and alignment modules will also be added in order to support classification tools that require a contig or alignment file as input, respectively. We welcome input by the user community, including contributions to continue the development of META, additional dockerized classifiers, and even additional containment schemes which may scale to other use cases in the High Performance Computing (HPC) community.

## Material and methods

### Custom reference sequence sets

In order to directly compare the performance of each classifier, a custom database was generated from the same set of reference organisms. This database included all archaea (347), bacteria (16,678), fungi (11), and viral (8,999) assemblies available on NCBI RefSeq that had a “latest” version status and an assembly classified as a complete genome, downloaded 1 October 2020. For the environmental bacteria use case, the *Bacillus atrophaeus* subsp. *globigii* taxID was purposefully omitted in the custom reference database. The approximate total size of the custom nucleic acid database was 69 GB, and the protein sequence database was 24 GB.

### Classifier inclusion criteria

Seven critical features were analyzed for each metagenomic classifier in order to down-select the final list for inclusion in the initial release of META: open source accessibility, availability on the Bioconda software package manager, date of last update, local data storage options, custom database input options, classification strategy, and analysis type. To be included in the META system, a classifier had to be open source and available on Bioconda, a software package manager whose support increases ease of deployment and integration of the classifier. The presence of a classifier on Bioconda also implies that the tool is well-accepted and tested in the broader bioinformatics community. Recent updates were required to ensure that only actively maintained classifiers were selected, and as META requires classifiers to be run locally, any tool deployed only in a cloud-based environment was filtered out.

As mentioned above, in order to directly compare performance across classifiers, each tool had to allow for the use of a custom database, and due to the limited specificity of classifications based on marker genes, particularly 16S rRNA, all classifiers utilizing this strategy were excluded. This study aimed to review metagenomic classifier tools only, meaning other sequencing data analysis tool (e.g., assemblers, aligners, wrappers, etc.) were excluded, but could be included in future iterations of META in order to test and evaluate more complex analysis pipelines.

### System architecture and workflow

All META files and installation instructions are openly available on GitHub at https://github.com/JHUAPL/meta-system, and https://github.com/JHUAPL/meta-simulator. For users already running Docker, containers of META are available at https://quay.io/repository/jhuapl/meta_system, and https://quay.io/repository/jhuapl/meta_simulator. META is designed to run as a local web server based on Python Flask, accessible *via* restful API or user interface optimized for Google Chrome and Firefox using the Vue. js framework (Flask Documentation, 2020; Vue.js, 2020) (Figure 1A). The architecture leverages Bioconda and BioContainers, Docker container versions of the most commonly used metagenomic classifiers, in order to provide consistent deployment (Merkel, 2014; Gruening et al., 2018; Grüning et al., 2018; BioContainers, 2020). Docker allows for the widest possible utility for those trying to optimize metagenomics workflows for lower resource systems. In addition, it remains the most widespread for existing classifier containerization, and so provides the best opportunity for broader comparisons of different strategies. Lastly, Docker containers can be imported into other containerization utilities such as Singularity (https://docs.sylabs.io/guides/3.5/user-guide/index.html). This allows META to easily incorporate new metagenomic classifiers or update older ones, providing a modular template for integration *via* a standard META YAML description (The Official YAML Web Site, 2020).

Towards interoperability, it was necessary to identify tool-specific commands that executed the four basic stages of metagenomic classification: download, build, classify, and report. This allowed for simultaneous processing of the same dataset by multiple classifiers without requiring individual inputs for each tool selected by the user. The following assumptions were made about all tools integrated with META; the tool database relies on a set of reference nucleotide or protein sequences for classification, and the tool can perform an analysis that provides output that allows for relative abundance calculations to be produced. These assumptions led to the following definitions within the META ecosystem: 1) download: the command that downloads the set of reference nucleotide or protein sequences. Depending on the use-case, the set of reference sequences may already be present on the host machine. This is likely the case for those seeking to build custom reference databases. Currently, this command isn’t automatically executed within the META system. 2) Build: the command’s that build the database indices and relevant files from the set of downloaded reference sequences. An example of this is “kraken-build --db kraken_db --build”. Currently, this command isn’t automatically executed within the META system. 3) Classify: the command that runs classification of sequence input. Classification analysis can be performed using several algorithms, including, but not limited to, distance metrics, string comparisons, or expectation maximizations (EMs). The command ideally contains an argument that specifies an output directory or output file path. An example of this is “kraken --db $db --output $output $input”. 4) Report: the command pro that formats outputs. An example of this is “kraken-report --db $db $output”. An additional command may be necessary to rename the formatted report to “<tool_name>.report”. Some tools may not bundle a reporting utility, in which case, the META report file will be generated in the classify stage, and no report command needs to be identified. Notice that a set of installation commands aren’t required. This highlights the advantage of using BioContainer Docker images for deployment, as it doesn’t require end users to install the tool and its dependencies prior to using it.

When a user is ready to process data using META, they must first submit a request in Mode 1 or Mode 2. To run a comparison using Mode 1 (*in silico* generated reads), an abundance profile must be provided in the form of a 3-column tab-separated variables (TSV) file. This file includes 1) the taxonomic ID (taxID) of the organisms making up the metagenomic community that will be simulated, 2) the relative abundance of the associated taxID (abundance must sum to a total of 1.000000), and 3) whether the taxID should 1) or shouldn’t 0) be considered in the relative abundance evaluation calculations. Users may opt to exclude certain taxID for abundance profile generation if the reads map to background genomic material or organisms of low interest. All input taxIDs in this file should map to an organism with a reference genome in NCBI’s RefSeq database, and a job will terminate and return an error message if a reference genome cannot be found for one or more taxIDs in an input TSV file. To run a comparison using Mode 2, the user must only provide a FASTQ file. All other parameters available to the user may be selected *via* check boxes on the job submission page, which include available classifiers to run and, for Mode 1 only, which read type(s) will be simulated for the provided abundance profile.

A META analytics workflow is automatically generated upon user request. Workflow components include simulation, classification, and evaluation modules that are processed serially. In Mode 1, for every simulated read type, there is an associated simulation module, and for every metagenomic classifier selected, there is an associated classification module. In Mode 2, the simulation module is excluded. In all modes, every FASTQ file has an associated evaluation module for computing metrics across all selected classifiers. Example workflows can be seen in Figure 1B. By distilling the workflow to these three modules (simulate, classify, evaluate) and four command types (download, build, classify, report), META prevents the “decision paralysis” inherent to more complex workflow environments and description languages (Leipzig, 2017; Perez-RiverolMoreno, 2020).

When a job is submitted, the user may view currently running jobs, and may select or download completed job reports for viewing or additional analysis. Most tables, graphs, and charts available in the report are fully interactive, sortable, and tips are provided where relevant. An additional “quick answers” menu, in the form of Frequently Asked Questions (FAQs) is available in order to guide the selection of classifier’s based on a particular metric of interest. When a FAQ is selected, this function automatically adjusts the data outputs to answer that specific question. Examples of questions include: “Which species has the highest L2?”, or “Which species and classifier combination has the largest AUPRC?” (Supplementary Figure S5).

### Evaluation and visualization

In Mode 1, the presence of known user-defined abundance profiles allows for direct comparison of classifier performance to ground truth values. This comparison is based on the ratio of the area under the precision recall curve (AUPRC) and the Euclidean distance between the user-defined abundance profile and the predicted abundance profile (L2) for each tool. These metrics are utilized because they are complementary as AUPRC is more sensitive to low abundance taxa, while L2 is more sensitive to high abundance taxa (Ye et al., 2019). For AUPRC, each point on the curve represents the precision and recall scores at a specific abundance threshold. Abundance thresholds from 0 to 1 are used to generate the full curve is generated, and the area calculated. The L2 metric is based on the pairwise Euclidean distance between the ground truth and classifier output taxa abundance vectors. Finally, a META score (referred to as metascore) is calculated from AUPRC and L2 by the following formula:

$$metascore=\left(\left(\left(AUPRC+1\right)/\left(L2/sqrt\left(2\right)\right)+1\right)\right)-0.5/1.5$$

Dividing L2 by its maximum value [sqrt (2)] normalizes the range to that of AURPC (0–1). The addition of one to both metrics before taking their ratio bounds the ratio’s range from to 1/2–2. Finally, subtracting .5 from the ratio then dividing by 1.5 adjusts the range of the metascore to 0–1. The metascore allows a user to see a quantitative, overall value that represents the combined performance of abundance calculation and taxonomic ID for a classifier and sequencer combination on a given mock metagenomic community (the user-defined TSV).

Classifier performance characteristics reported for both Mode 1 and 2 include a scatter/half-violin plot with a consensus call table, a parallel coordinates plot of resource utilization that includes CPU time, wall-clock time, and peak memory usage, and a sunburst plot of abundance that may be scaled and colored on various parameters to assist in data exploration. Visualizations were built using d3. js alongside the Vue. js frontend framework (MichaelVadim and Jeffrey, 2011; Vue.js, 2020). Each visualization is interactive and dynamic based upon user input and parameters attributed to a job. All tables available on the report page may be sorted and filtered and are linked to a single visualization.

The scatter/half-violin plot is designed to show the distribution of abundance calls across all classifiers for any number of read types (Mode 1 only) and specified ranks. Users may directly filter on any of these parameters within the plot by choosing from a dropdown menu or by zooming into a *y*-axis region. A table is also provided to display the specific point on the scatter plot that is attributed to a particular taxID upon cursor hover-over. An abundance thresholding distribution is provided to allow adjustment of the range of desired abundances to display.

Resource metrics including CPU time, wall-clock time, and peak memory usage for all selected classifiers (and read type for Mode 1) are plotted in a parallel coordinates plot. Each *y*-axis attributed to a metric is brush able to filter out undesired entries and is transferable (left-right) across the plot’s space for custom organization. Each line is hoverable to provide more information for that specific entry. A table is also provided that is directly linked to each line of the plot that is essential for identifying the best performing classifier-read type combination *via* metric sorting.

Finally, the sunburst provides a hierarchical representation of all taxIDs from super kingdom to strain ranks for a given classifier (and read type for Mode 1). Each slice size is based on the size of the abundance call for a given slice relative to the parent taxID and all sibling slices. Each slice is directly linked to an entry in a table and when clicked (table entry or slice), the plot dynamically updates to hide all ranks higher than the specified slice’s as well as all sibling slices at that rank. Color coding is selectable, and is based on either rank (Modes 1 and 2, default for Mode 2) or relative deviation from ground truth (input) abundance (mode 1 only, default for Mode 1; Supplemental Figure S6). Users may update the sunburst at any time with a dropdown selection of either the selected classifier and/or read type. An abundance threshold is also provided to allow users to adjust what range of reported abundances are to be observed in the plot, which is useful for classifiers that may call many unique taxIDs at exceedingly low abundances.

### Experimental use-case scenario 1: Pathogenic target in mixed environmental background

Use-case 1 was composed of equal genome copies from ten bacteria; *Bacillus anthracis* strain Ames (the “target” pathogenic organism), two taxonomic near neighbors of *B. anthracis*, and seven phylogenetically separate environmental organisms that are also represented in the ATCC environmental microbiome standard set. Live *Bacillus anthracis* was acquired from BEI Resources (NR-411), while the near neighbors *Bacillus anthracis* strain Sterne UT238 and *Bacillus globigii* were grown from in-house stocks. All cells were streaked on TSA plates and incubated for 48 h at 37°C. Colonies were selected from the plates and processed for nucleic acids using the DNeasy Blood and Tissue kit (Qiagen 69504). All other bacterial nucleic acids were obtained directly from ATCC as lyophilized reagents using the following catalog numbers: *Acinetobacter baumannii* (17978), *Bacteroides vulgatus* (8482), *Bifidobacterium adolescentis* (15703), *Clostridium beijerinckii* (35702), *Cutibacterium acnes* (11828), *Rhodobacter sphaeroides* (17029), *and Staphylococcus epidermidis* (12228). Lyophilized nucleic acids were resuspended in nuclease-free water prior to combining. Organism nucleic acids were combined to generate four unique sample types for evaluation: 1) *B. anthracis* alone, 2) *B. anthracis* and near-neighbors, 3) 7 background organisms, and 4) all ten components. For each sample type, all organisms were spiked at equal genome copy per organism with an expected metagenomic profile of equal relative taxonomic abundances. Interestingly, the performance of most classifiers is a function of the taxonomic relative abundance, and the sequence relative abundance, since few organisms are exactly the same size. This bias between taxa and sequence relative abundance has recently been reiterated as a concern in the community by Sun et al., (2021). However, this conflict would still be represented in real world samples, and so any benchmarking of classifier performance on a given sample set would represent this complexity.

### Experimental use-case scenario 2: Pathogenic target in host background

Use case 2 was composed of a viral pathogenic target present in a host cell background. Vaccinia virus strain MVA was acquired from BEI (NR-1) and was selected as the target viral pathogen due to ease of propagation, its DNA genome, and its similarity to the biothreat agent smallpox. VACV was grown in chicken (*Gallus gallus*) embryo fibroblast cells (ATCC CRL-1590) according to vendor recommendations. Briefly, cells were grown to 80% confluence at 37°C and 5% CO_2_ in growth media containing DMEM supplemented with 5% fetal bovine serum (FBS) and 5% tryptose phosphate broth, and infected at a multiplicity of infection (MOI) of 0.05 in inoculation media containing DMEM only. Following a 1-h incubation period, inoculation media was removed and replaced with growth media. Three days post-infection, cells were scraped, centrifuged at 1,200 x g for 10 min at 4°C, and resuspended in DMEM supplemented with 2% FBS. Cells were lysed using 3 freeze-thaw cycles and the final lysate was sonicated in ice water. Aliquots were stored at −80°C. Nucleic acid was isolated from both viral lysates and host cell lysates using the Qiagen QIAmp DNA Mini kit (51304). Viral and host nucleic acid was combined to generate four unique samples types: 1) host cells alone, 2) neat viral lysate, 3) 1:10 dilution of viral lysate in host background, and 4) 1:100 dilution of viral lysate in host background. Exact abundance of viral nucleic acid relative to host in samples 2–4 wasn’t known prior to sequencing.

### Illumina and oxford nanopore technologies sequencing

Illumina MiSeq libraries were generated using Illumina Nextera XT library preparation kits (cat# FC-131-1096). All thirty libraries generated from both use cases were multiplexed together on a 2 × 300 paired end sequencing run using a 600 cycle MiSeq reagent kit (cat# MS-102-3003). All samples and use cases were sequenced as triplicates to achieve technical replication. Positive controls consisted of pathogen, host extraction, or organism-only controls, while negative controls consisted of Nextera library with and without barcodes and barcodes only. Barcodes for previous runs were checked and filtered during analysis for standard contamination avoidance. All Oxford Nanopore Technologies (ONT) libraries were generated using Rapid barcoding sequencing kits (cat# SQK-RBK004). Sample libraries were multiplexed up to five samples per run and sequenced on three separate sequencing runs on an Oxford Nanopore GridION Sequencer using ONT R9 flowcells (cat# FLO-MIN106D). Positive controls consisted of pathogen, host extraction, or organism-only controls, while negative controls consisted of replicates of Rapid libraries and barcodes. For further contamination avoidance, all nanopore flow cells were used only once (i.e., no flow cells were washed and re-used). Barcodes for previous runs were also check and filtered, and resulting files used in analyses.

## Data Availability

The META system is available on the GitHub page of JHU/APL: https://github.com/JHUAPL/meta-system. Full installation of the META system includes running the open-source software from a Docker container. For users already running Docker, containers of META may be accessed at https://quay.io/repository/jhuapl/meta_system, and https://quay.io/repository/jhuapl/meta_simulator. Additional META software dependencies include Node 10+ and NPM 6+ (front-end), and Python 3.7+ 12 (back-end). The softwarewas developed on an Ubuntu 18.04 virtual machine containing 32 CPU cores, 512 GB RAM, and 10 TB disk space. Illumina and ONT data is available on NCBI’s SRA, accession PRJNA904684, at the following link: https://www.ncbi.nlm.nih.gov/bioproject/904684.

## Supplementary material

Supplementary Figure S1 Use case 1 output for AUPRC only. Classifier performance from *in vitro* data sets is illustrated using the AUPRC metric from superkingdom to strain taxonomic ranking. All twelve available classifiers were run using data generated from Illumina MiSeq and the ONT R9 flowcell. *N*=3.

Supplementary Figure S2 Use case 1 output for L2 only. Classifier performance from *in vitro* data sets is illustrated using the L2 metric from superkingdom to strain taxonomic ranking. All twelve available classifiers were run using data generated from Illumina MiSeq and the ONT R9 flowcell. *N*=3.

Supplementary Figure S3 Use case 2 output for AUPRC only. Classifier performance from *in vitro* data sets is illustrated using the AUPRC metric from superkingdom to strain taxonomic ranking. All twelve available classifiers were run using data generated from Illumina MiSeq and the ONT R9 flowcell. *N*=3.

Supplementary Figure S4 Use case 2 output for L2 only. Classifier performance from *in vitro* data sets is illustrated using the L2 metric from superkingdom to strain taxonomic ranking. All twelve available classifiers were run using data generated from Illumina MiSeq and the ONT R9 flowcell. *N*=3.

Supplementary Figure S5 Graphical User Interface Visualization from META, illustrating the drop-down menu of FAQs. Selection of an FAQ automatically adjusts the data representation to emphasize how the data addresses the specific question.

Supplementary Figure S6 Graphical User Interface Visualization from META, illustrating how the deviation from ground truth can be visualized by either a sunburst plot, or a table.

## References

[B1] Afgan E. Baker D. Batut B. van den Beek M. Bouvier D. Cech M. (2018). The Galaxy platform for accessible, reproducible and collaborative biomedical analyses: 2018 update. Nucleic Acids Res. 46, W537–W544. 10.1093/nar/gky379 29790989 PMC6030816

[B2] BioContainers BioContainers Community including registry, documentation, specification. Avaliable at: https://biocontainers.pro/#/ (Accessed on Sept 16, 2020).

[B3] Bray N. L. Pimentel H. Melsted P. Pachter L. (2016). Near-optimal probabilistic RNA-seq quantification. Nat. Biotechnol. 34, 525–527. 10.1038/nbt.3519 27043002

[B4] Breitwieser F. P. Baker D. N. Salzberg S. L. (2018). KrakenUniq: Confident and fast metagenomics classification using unique k-mer counts. Genome Biol. 19, 198. 10.1186/s13059-018-1568-0 30445993 PMC6238331

[B5] Buchfink B. Xie C. Huson D. H. (2015). Fast and sensitive protein alignment using DIAMOND. Nat. Methods 12, 59–60. 10.1038/nmeth.3176 25402007

[B6] Flask Documentation Welcome to Flask — Flask documentation (1.1.x). Avaliable at: https://flask.palletsprojects.com/en/1.1.x/ (Accessed on Sept 16, 2020).

[B7] GA4GH Enabling responsible genomic data sharing for the benefit of human health. Avaliable at: https://www.ga4gh.org/ (Accessed on Sept 14, 2020).

[B8] Gourlé H. Karlsson-Lindsjö O. Hayer J. Bongcam-Rudloff E. (2019). Simulating Illumina metagenomic data with InSilicoSeq. Bioinformatics 35, 521–522. 10.1093/bioinformatics/bty630 30016412 PMC6361232

[B9] Gruening B. Sallou O. Moreno P. da Veiga Leprevost F. Menager H. Sondergaard D. (2018). Recommendations for the packaging and containerizing of bioinformatics software. F1000Res 7, 742. 10.12688/f1000research.15140.2 PMC6738188 31543945

[B10] Grüning B. Dale R. Sjodin A. Chapman B. A. Rowe J. Renan V. (2018). Bioconda: Sustainable and comprehensive software distribution for the life sciences. Nat. Methods 15, 475–476. 10.1038/s41592-018-0046-7 29967506 PMC11070151

[B11] Kim D. Song L. Breitwieser F. P. Salzberg S. L. (2016). Centrifuge: Rapid and sensitive classification of metagenomic sequences. Genome Res. 26, 1721–1729. 10.1101/gr.210641.116 27852649 PMC5131823

[B12] Leipzig J. (2017). A review of bioinformatic pipeline frameworks. Brief. Bioinform 18, 530–536. 10.1093/bib/bbw020 27013646 PMC5429012

[B13] Li Y. Han R. Bi C. Li M. Wang S. Gao X. (2018). DeepSimulator: A deep simulator for nanopore sequencing. Bioinformatics 34, 2899–2908. 10.1093/bioinformatics/bty223 29659695 PMC6129308

[B14] Lu J. Breitwieser F. P. Thielen P. Salzberg S. L. (2017). Bracken: Estimating species abundance in metagenomics data. PeerJ Comput. Sci. 3, e104. 10.7717/peerj-cs.104 PMC12016282 40271438

[B15] Menzel P. Ng K. L. Krogh A. (2016). Fast and sensitive taxonomic classification for metagenomics with Kaiju. Nat. Commun. 7, 11257–11259. 10.1038/ncomms11257 27071849 PMC4833860

[B16] Merkel D. (2014). Docker: Lightweight linux containers for consistent development and deployment. Linux J. 2014. Article (2) March Avaliable at: https://dl.acm.org/doi/abs/10.5555/2600239.2600241 (Accessed on Sept 14, 2020).

[B17] Meyer F. Bremges A. Belmann P. Janssen S. McHardy A. C. Koslicki D. (2019). Assessing taxonomic metagenome profilers with OPAL. Genome Biol. 20, 51. 10.1186/s13059-019-1646-y 30832730 PMC6398228

[B18] MichaelVadim B. O. Jeffrey H. (2011). “D3 data-driven documents,” in IEEE transactions on visualization and computer graphics. 10.1109/TVCG.2011.185 22034350

[B19] Ondov B. D. Starrett G. J. Sappington A. Kostic A. Koren S. Buck C. B. (2019). Mash screen: High-throughput sequence containment estimation for genome discovery. Genome Biol. 20, 232. 10.1186/s13059-019-1841-x 31690338 PMC6833257

[B20] Ounit R. Wanamaker S. Close T. J. Lonardi S. (2015). Clark: Fast and accurate classification of metagenomic and genomic sequences using discriminative k-mers. BMC Genomics 16, 236. 10.1186/s12864-015-1419-2 25879410 PMC4428112

[B21] Park S. T. Kim J. (2016). Trends in next-generation sequencing and a new era for whole genome sequencing. Int. Neurourol. J. 20, S76–S83. 10.5213/inj.1632742.371 27915479 PMC5169091

[B22] Perez-Riverol Y. Moreno P. (2020). Scalable data analysis in proteomics and metabolomics using BioContainers and workflows engines. Proteomics 20 (9), 1900147. 10.1002/pmic.201900147 PMC7613303 31657527

[B23] Steinegger M. Söding J. (2017). MMseqs2 enables sensitive protein sequence searching for the analysis of massive data sets. Nat. Biotechnol. 35, 1026–1028. 10.1038/nbt.3988 29035372

[B24] Sun Z. Huang S. Zhang M. Zhu Q. Haiminen N. Carrieri A. P. (2021). Challenges in benchmarking metagenomic profilers. Nat. Methods 18, 618–626. 10.1038/s41592-021-01141-3 33986544 PMC8184642

[B25] The Official YAML Web Site The official YAML web site. Avaliable at: https://yaml.org/ (Accessed on Sept 16, 2020).

[B26] Vue.js The progressive JavaScript framework. Avaliable at: https://vuejs.org/ (Accessed on Sept 16, 2020).

[B27] Wood D. E. Lu J. Langmead B. (2019). Improved metagenomic analysis with Kraken 2. Genome Biol. 20, 257. 10.1186/s13059-019-1891-0 31779668 PMC6883579

[B28] Wood D. E. Salzberg S. L. (2014). Kraken: Ultrafast metagenomic sequence classification using exact alignments. Genome Biol. 15, R46. 10.1186/gb-2014-15-3-r46 24580807 PMC4053813

[B29] Ye S. H. Siddle K. J. Park D. J. Sabeti P. C. (2019). Benchmarking metagenomics tools for taxonomic classification. Cell 178, 779–794. 10.1016/j.cell.2019.07.010 31398336 PMC6716367

[B30] Ying C. Weicai Y. Yongdong Z. Yuesheng X. (2015). High speed BLASTN: An accelerated MegaBLAST search tool. Nucleic acids Res. 43, 7762–7768. 10.1093/nar/gkv784 26250111 PMC4652774

