# Supplementary figures and images for: The META tool optimizes metagenomic analyses across sequencing platforms and classifiers
Source: Front Bioinform. 2023 Jan 6;2:969247. doi: 10.3389/fbinf.2022.969247 (PMC9852826; doi:10.3389/fbinf.2022.969247)

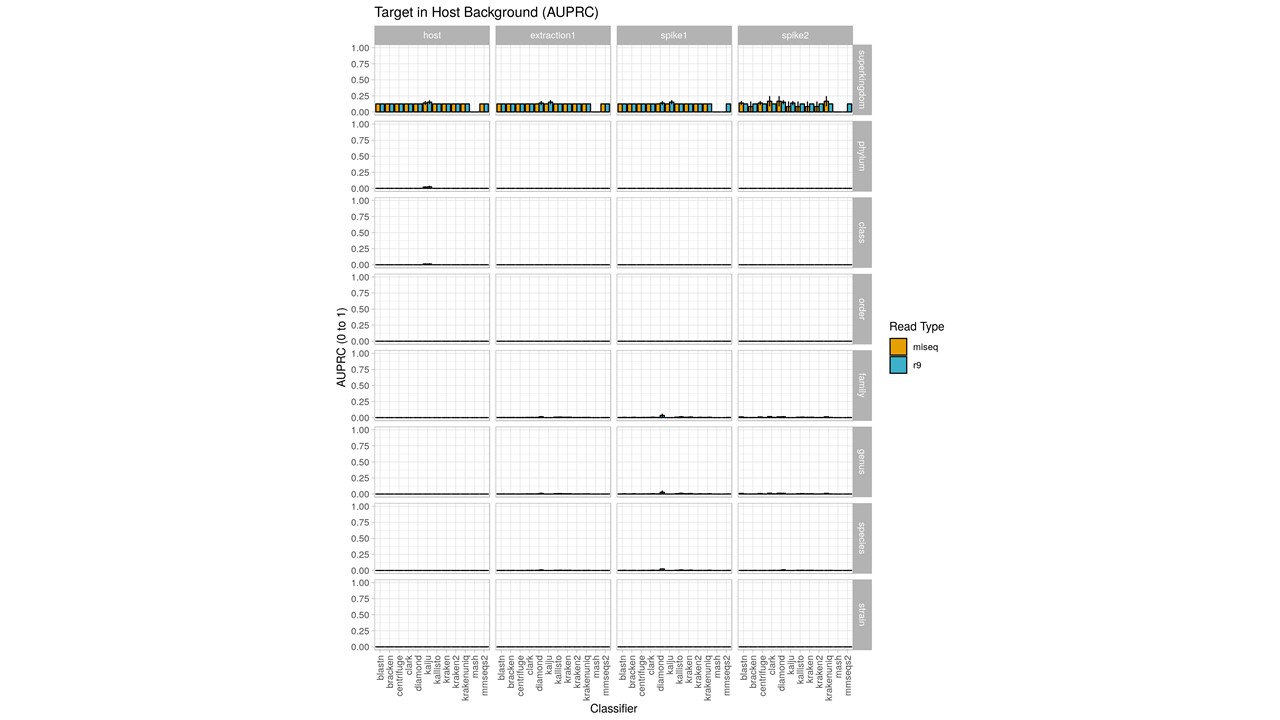

Supplement: Supplementary file 2 [file Image3.JPEG]

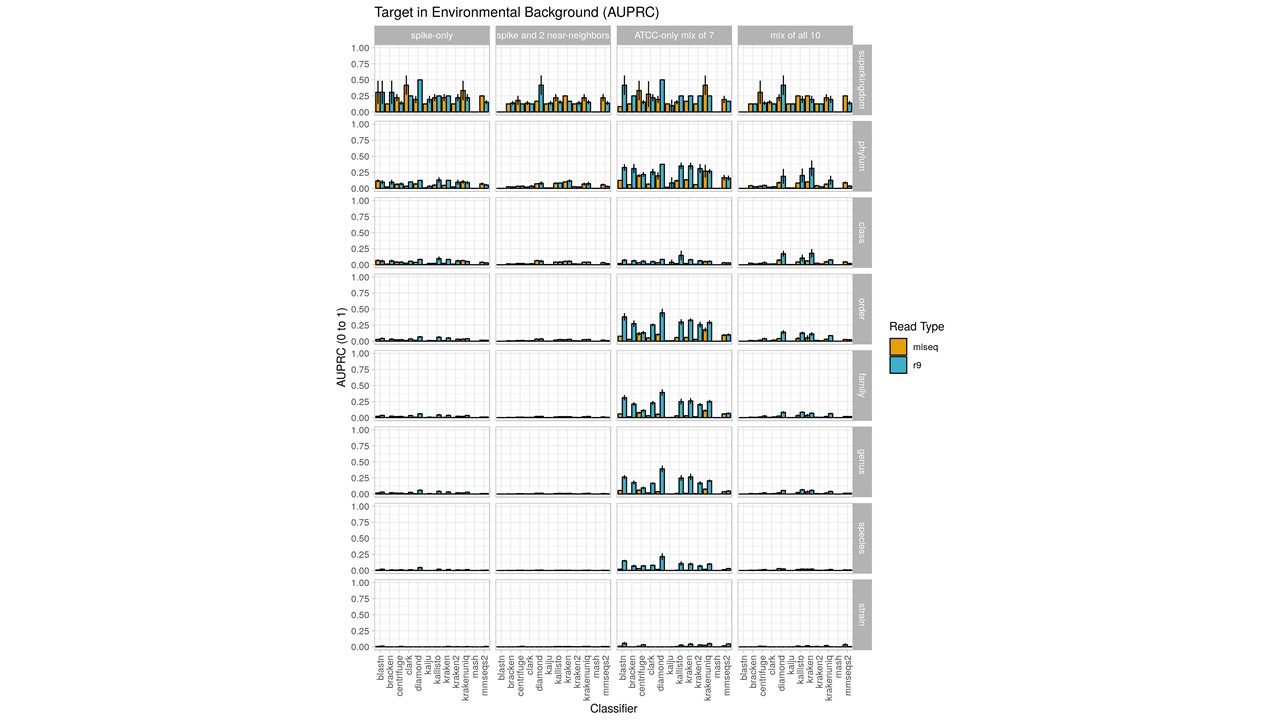

Supplement: Supplementary file 4 [file Image1.JPEG]

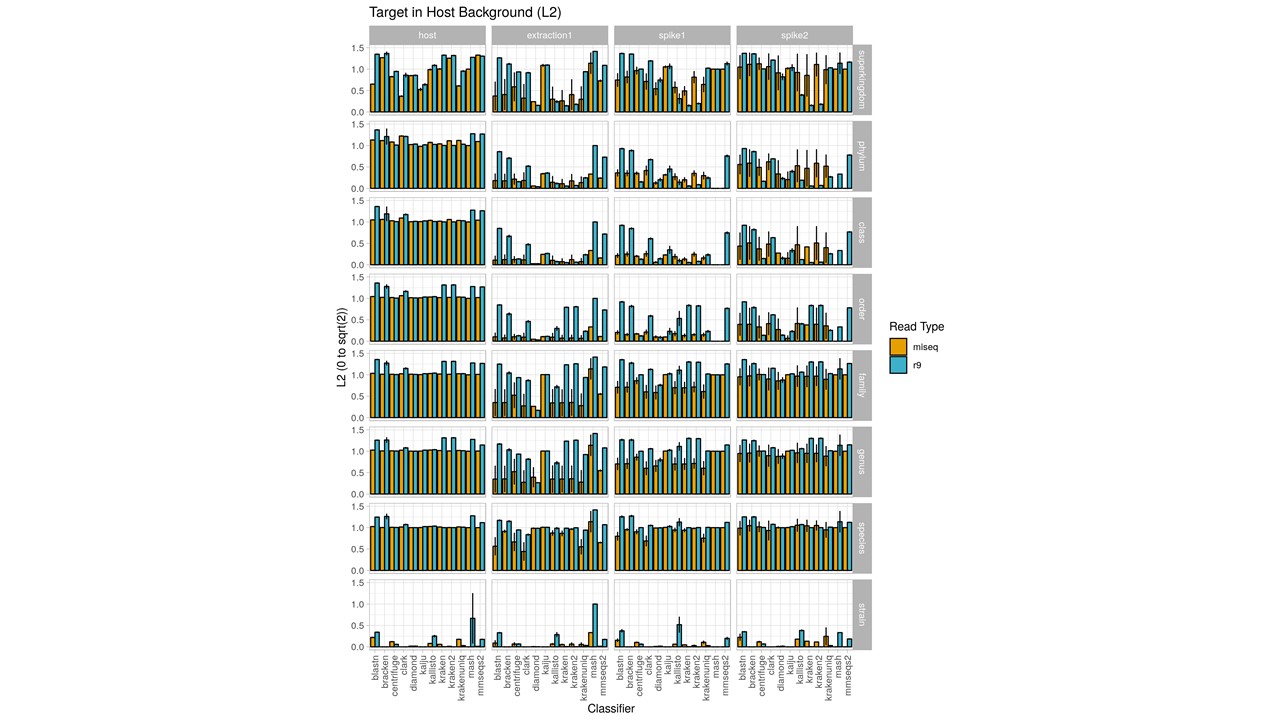

Supplement: Supplementary file 5 [file Image4.JPEG]

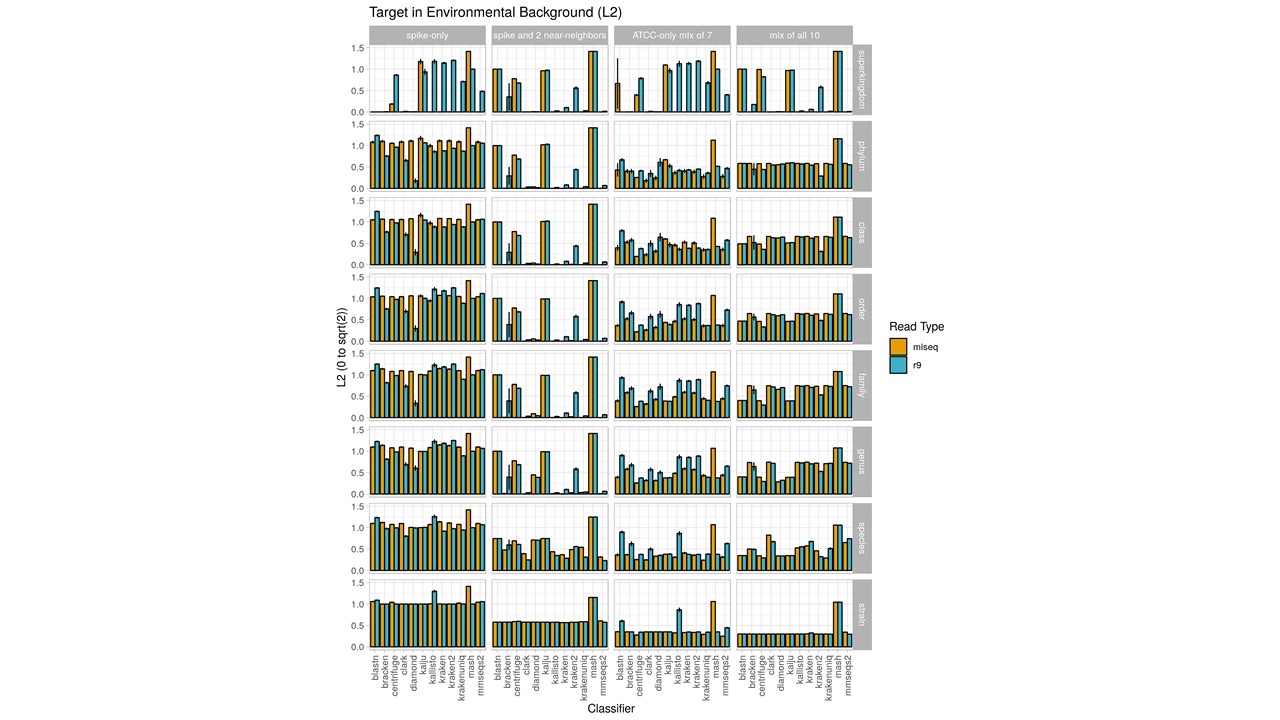

Supplement: Supplementary file 6 [file Image2.JPEG]

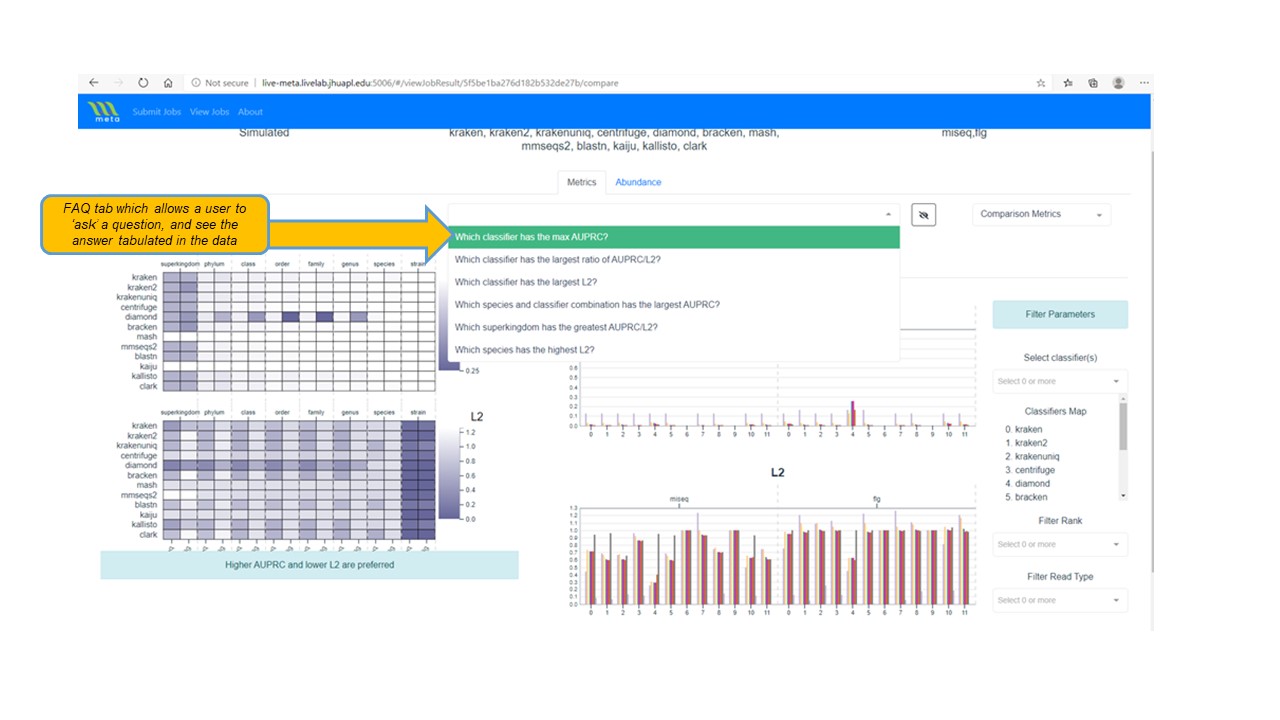

Supplement: Supplementary file 7 [file Image5.JPEG]

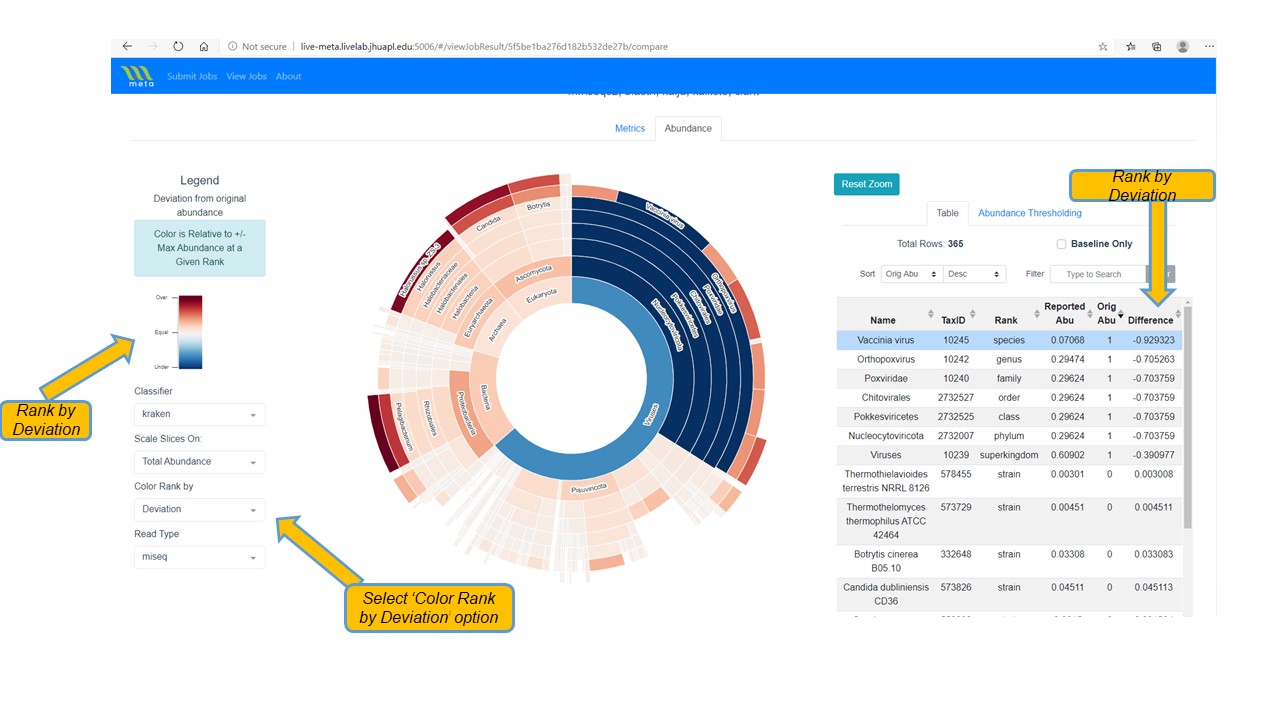

Supplement: Supplementary file 10 [file Image6.JPEG]
